# Assessment for antibiotic resistance in *Helicobacter pylori*: A practical and interpretable machine learning model based on genome-wide genetic variation

**DOI:** 10.1080/21505594.2025.2481503

**Published:** 2025-03-21

**Authors:** Yingying Wang, Shuwen Zheng, Rui Guo, Yanke Li, Honghao Yin, Xunan Qiu, Jijun Chen, Chuxuan Ni, Yuan Yuan, Yuehua Gong

**Affiliations:** a Tumor Etiology and Screening Department of Cancer Institute and General Surgery, The First Hospital of China Medical University, Shenyang, China; b Key Laboratory of Cancer Etiology and Prevention in Liaoning Education Department, The First Hospital of China Medical University, Shenyang, China; c Key Laboratory of GI Cancer Etiology and Prevention in Liaoning Province, The First Hospital of China Medical University, Shenyang, China

**Keywords:** *Helicobacter pylori*, machine learning, antibiotic resistance, interpretable model, web calculator

## Abstract

*Helicobacter pylori* (*H. pylori*) antibiotic resistance poses a global health threat. Accurate identification of antibiotic resistant strains is essential for the control of infection. In the present study, our goal is to leverage the whole-genome data of *H. pylori* to develop practical and interpretable machine learning (ML) models for comprehensive antibiotic resistance assessment. A total of 296 *H. pylori* isolates with genome-wide data were downloaded from the Bacterial and Viral Bioinformatics Resource Center (BV-BRC) and the National Center for Biotechnology Information (NCBI) databases. By training ML models on feature sets of single nucleotide polymorphisms from SNP calling (SNPs-1), antibiotic-resistance SNP annotated by the Comprehensive Antibiotic Resistance Database (SNPs-2), gene presence or absence (GPA), we generated predictive models for four antibiotics and multidrug-resistance (MDR). Among them, the models that combined SNPs-1, SNPs-2, and GPA data demonstrated the best performance, with the eXtreme Gradient Boosting (XGBoost) consistently outperforming others. And then we utilized the SHapley Additive exPlanations (SHAP) method to interpret the ML models. Furthermore, a free web application for the MDR model was deployed to the GitHub repository (https://H.pylori/MDR/App/). Our study demonstrated the promise of employing whole-genome data in conjunction with ML algorithms to forecast *H. pylori* antibiotic resistance. In the future, the application of this approach for predicting *H. pylori* antibiotic resistance would hold the potential to mitigate the empiric administration.

## Introduction

*Helicobacter pylori* (*H. pylori*) colonization is a common chronic bacterial infection, affecting over half the global population [1]. Numerous randomized controlled trials and cohort studies have shown that eradicating *H. pylori* can significantly reduce the risk of gastric cancer, making it the primary modifiable risk factor for preventing gastric disease [2–5]. Over the past 40 years, metronidazole (MTZ), clarithromycin (CLR), levofloxacin (LEV), amoxicillin (AMX), and tetracycline (TET) have been commonly used to eradicate *H. pylori*, with various strategies proposed [6,7]. However, global reviews and meta-analyses have shown a decline in *H. pylori* eradication rates and an increase in antibiotic resistance [8,9]. Therefore, it is crucial to understand the antimicrobial resistance profiles of *H. pylori* and guide eradication treatment based on antibiotic susceptibility testing (AST).

Currently, drug susceptibility-guided personalized therapy for *H. pylori* infection involves two main methods: Culture-dependent phenotypic techniques (e.g. agar dilution and E-test) and molecular methods based on resistance mutations (e.g. PCR). Nevertheless, these strategies have some limitations that hinder their clinical applicability. Culture-based methods are time-consuming, taking about 2–3 weeks, and require specialized facilities and trained technicians. PCR-based methods are limited in the types of antibiotics they can detect, being primarily suitable for predicting resistance to CLR and LEV [10–12]. For other common antibiotics, genotypic detection methods are not available due to the lack of identified resistance-related molecules. Moreover, such methods are insufficient for discovering new or rare resistance characteristics [13].

Fortunately, the genomics era has provided an extensive collection of strain-specific whole-genome sequencing (WGS) for various pathogenic bacteria, including *H. pylori*. This data enables the use of big data informatics to investigate antimicrobial resistance. However, manual analysis of large-scale datasets is time-consuming, resource-intensive, and prone to subjectivity. In contrast, machine learning (ML) can efficiently process large, high-dimensional, and unstructured data, offering objective analysis and continuous improvement by learning from new data. The integration of clinical antimicrobial resistance data with strain-specific genome sequences has opened new avenues for studying antibiotic resistance using ML and big data methodologies. This approach has exhibited promise within pathogenic bacteria contexts, including *Escherichia coli*, *Mycobacterium tuberculosis*, *Salmonella*, and *Pseudomonas aeruginosa* [14–17].

However, the “black-box” nature of ML algorithms can make it challenging to explain their predictions. This lack of interpretability limits the use of powerful ML methods in medical decision support. To address this, the SHapley Additive exPlanation (SHAP) was developed. SHAP is a Shapley value-based method that equitably distributes the contribution of individual features in ML model predictions. It is additive, fair, model-independent, and can detail the impact of each feature on the predictions. This enhances model transparency and interpretability, providing a deeper understanding of the complex relationships between features and predictions.

In this study, we integrated WGS data and ML techniques, utilizing comprehensive genomic information of *H. pylori* to predict resistance phenotypes. By visualizing with SHAP values, the interpretability of the models was enhanced, and the key features influencing the prediction model were identified. Ultimately, we developed a free and user-friendly web calculator to help clinicians and healthcare professionals make informed decisions about *H. pylori* eradication and individualized treatment.

## Materials and methods

### WGS data collection of *H. pylori* isolates

296 *H. pylori* WGS data in FASTA format were downloaded from the Bacterial and Viral Bioinformatics Resource Center (BV-BRC) and the National Center for Biotechnology Information (NCBI) databases by June 2024. The selection criteria were as follows: (i) the level of genome assembly must be complete, (ii) the bacterium must be isolated from a human host, (iii) the sequencing depth of at least 100x, (iv) there must be available resistance information for at least three or more antibiotics, including LEV, MTZ, CLR, AMX, and TET. Subsequently, we re-annotated the resistance of all strains with reference to the European Committee on Antimicrobial Susceptibility Testing (EUCAST version 13.1): a strain was deemed resistant if its minimum inhibitory concentration (MIC) exceeded 8 μg/ml for MTZ, 0.5 μg/ml for CLR, 1 μg/ml for LEV, 0.125 μg/ml for AMX, and 1 μg/ml for TET. Moreover, strains exhibiting resistance to three or more antibiotics were designated as multidrug-resistant (MDR). Details of *H. pylori* WGS data were showed in Dataset EV1.

### Pan-genome construction and functional analysis

The genomes were first annotated using Prokka (v1.13) [18], and the pan-genome was constructed using Roary (v3.12.0) [19], a tool that rapidly builds large-scale pan genomes and identifies core genes and accessory genes, with a minimum blastp identity of 95% and a core definition threshold of 100%. A total of 9599 genes were identified, comprising 490 core genes and 9109 accessory genes, which together formed a binary matrix representing the presence-absence of genes (GPA). For functional analysis using eggNOG-mapper v2 [20], significant differences in abundance (at least 2-fold difference) between the core and accessory groups were considered.

### SNP calling and phylogenetic analysis

Variant calling, including single nucleotide polymorphisms (SNPs), insertions, deletions, multi-nucleotide polymorphisms, and complex variations, was performed using Snippy v4.6.0 with default settings (https://github.com/tseemann/snippy). The WGS data ensured comprehensive variant detection, yielding a dataset of 208,277 SNPs, which were summarized to construct the resistance matrix (SNPs-1). Core genome SNPs were identified using SNP-sites, and a core-SNP-based phylogenetic tree was constructed with FastTree v2.1.10 and visualized using iTOL. *H. pylori* 26695 (Genome assembly ID: GCF_000307795.1) was used as the reference genome.

### Antibiotic resistance-related SNPs annotation from CARD

The whole genomes of 296 *H. pylori* strains were annotated using the Resistance Gene Identifier (RGI v6.0.3) from the Comprehensive Antibiotic Resistance Database (CARD v3.2.9). The RGI tool was run in “loose” mode with the DIAMOND program to ensure comprehensive detection of resistance-associated mutations, including those of low homology. A total of 91 resistance-associated SNPs were identified and summarized to construct the resistance matrix (SNPs-2).

### Feature selection for ML model predictions

Model predictions were conducted using five different feature sets as inputs: 1) matrix of SNPs-1 from the whole genomics; 2) matrix of SNPs-2 annotated by CARD; 3) matrix of SNPs-1+SNPs-2; 4) matrix of GPA; and 5) matrix of combinations of SNPs-1+SNPs-2+GPA. The dataset was randomly divided into a training set (80%) and a validation set (20%). The Random Forest (RF, n_estimators = 500, max_depth = 6) algorithm was employed for initial feature selection, followed by feature standardization. Subsequently, the Least Absolute Shrinkage and Selection Operator (LASSO) with 10-fold cross-validation was applied, retaining only features with non-zero coefficients. To address the data imbalance issue in the training set, we employed the adaptive synthetic algorithm, which reduced error rates and improved accuracy.

### ML models development and validation

Several ML algorithms were applied. K-Nearest Neighbors (KNN): Classifies a data point based on the most common class among its k nearest neighbors. Logistic Regression (LR): A widely used classical linear prediction algorithm for classification tasks. Support Vector Machine (SVM): Identifies the optimal hyperplane to separate samples with different labels. RF: An ensemble method based on bagging, combining multiple decision trees through majority voting to achieve high accuracy and robust performance. Gradient Boosting Decision Trees (GBDT) and eXtreme Gradient Boosting (XGBoost): Boosting-based ensemble algorithms that enhance prediction accuracy and reduce classification error by combining multiple weak classifiers.

To optimize the models’ performance, we combined a ten-fold cross-validation grid search with conventional methods to determine the final hyperparameters. Each model’s performance was evaluated and compared using metrics like accuracy, precision, recall, F1-score, and the area under the curve (AUC). The best-performing model was selected as the final predictor based on the evaluation results.

The specific definitions of these parameters are as follows:

$$\begin{array}{c} \mathrm{Accuracy}=\frac{\mathrm{True}\;\mathrm{positives}\;+\;\mathrm{True}\;\mathrm{ne}gat\mathrm{ives}}{\mathrm{True}\;\mathrm{positives}\;+\;\mathrm{True}\;\mathrm{ne}gat\mathrm{ives}}\\ +\mathrm{False}\;\mathrm{positives}+\mathrm{False}\;\mathrm{negatives} \end{array}$$

$$\mathrm{Precision}=\frac{\mathrm{True}\;\mathrm{positives}}{\mathrm{True}\;\mathrm{positives}+\mathrm{False}\;\mathrm{positives}}$$

$$\mathrm{Recall}=\frac{\mathrm{True}\;\mathrm{positives}}{\mathrm{True}\;\mathrm{positives}+\mathrm{False}\;\mathrm{negatives}}$$

$$F1\_\mathrm{score}=\frac{2*\mathrm{Precision}*\mathrm{Recall}}{\mathrm{Precision}+\mathrm{Recall}}$$

### ML models interpretation and visualization

We employed SHAP (v0.39.0, available at https://github.com/slundberg/shap) to provide both local and global interpretability for the models, a game theory-based method that uses Shapley values to optimally allocate credit and estimate feature importance. Specifically, the SHAP summary plot illustrated feature importance and their relationships with the model output, while the SHAP force plot provided an intuitive visualization of how different features impact individual predictions. The entire process was implemented in Python (v3.9) using the scikit-learn library (https://scikit-learn.org/stable/).

### Web-based calculator deployment

The optimal model and the corresponding Python code were uploaded to the GitHub (https://github.com/H.pylori/MDR). A web calculator (https://H.pylori/MDR/App/) can be deployed by interlinking the Streamlit app with the GitHub repository.

## Results

### Distribution of antimicrobial resistance and phylogenetic analysis in 296 *H. pylori* isolates

**Figure 1.**
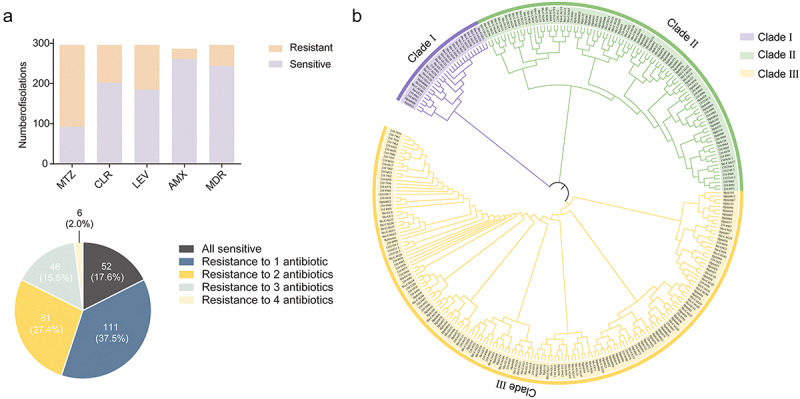
Antibiotic susceptibility overview and phylogenetic distribution of 296 *H. pylori* strains. (a) Antimicrobial susceptibility profiles after re-annotation of MTZ, CLR, LEV, and AMX according to European committee on antimicrobial susceptibility testing (EUCAST version 13.1). (b) SNP-based phylogenetic analysis of *H. pylori* strains. For each isolate, its sequence was mapped onto the *H. pylori* 26695 genome. Three major clades have been shown by free-hand shapes with different colors. Clade I included strains such as Shi-CAG1, Shi-GU7, and Hpfe033. Clade II contained strains like 210.8971, Hpfe080, and Shi-CSG15. Clade III included strains like Hpfe102, 210.7959 and 210.8942. Abbreviations: MTZ, metronidazole; CLR, clarithromycin; LEV, levofloxacin; AMX, amoxicillin; MDR, multidrug-resistant.

First, we analyzed the distribution of antimicrobial resistance among the 296 *H. pylori* strains (Supplementary Table S1). Resistance phenotype information was available for all strains with regard to MTZ, CLR, and LEV showing that 204 strains were resistant to MTZ, 94 strains were resistant to CLR and 111 strains were resistant to LEV. For AMX, and TET, resistance phenotype information was available for 287 and 172 strains, respectively. Among these, 26 strains were resistant to AMX, and 3 strains exhibited resistance to TET. Notably, 52 isolates were classified as MDR (Figure 1(a)). Due to the low resistance rate of TET, we collected insufficient positive samples for TET resistance. Therefore, we excluded this data from subsequent model construction to avoid unstable performance or biased predictions caused by sample imbalance. A total of 276 strains with age and sex information was presented in Supplementary Table S2. Apart from a potential correlation between gender and LEV sensitivity, there was no significant association among others.

Next, we analyzed the phylogenetic relationships of 296 *H. pylori* isolates using a phylogenetic tree based on the core SNPs. The isolates were divided into three clades (Figure 1(b)), which showed strains from China, the United States, Colombia, and Malaysia clustered together, indicating close genetic relationships.

### Pan-genome and functional analysis of 296 *H. pylori* isolates

**Figure 2.**
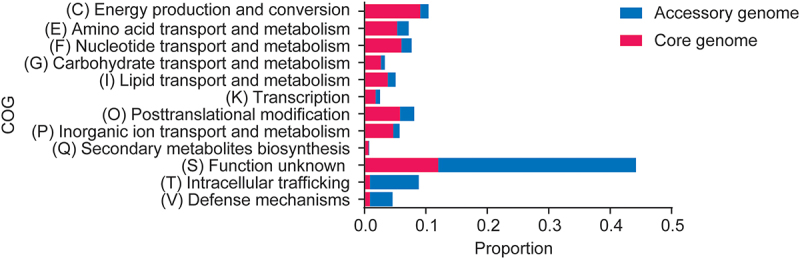
The functional analysis of COGs between the core and the accessory genomes. Estimated the COG percentage by dividing the COG number by the total number of gene clusters in the core or accessory genome. Only COGs differing by at least 2-fold between the core and accessory parts were included. Abbreviations: COG, the clusters of orthologous groups.

Pan-genome analysis of 296 *H. pylori* isolates revealed significant genetic distribution and variability (Supplemental Figure S1). The increase in orthologous gene clusters with the number of genomes analyzed indicated that *H. pylori* had an open pan-genome. Functional analysis of the Clusters of Orthologous Groups (COGs) revealed that the core genome was predominantly associated with crucial cellular processes. In contrast, the annotation of the accessory genome indicated that these gene clusters were typically linked to genetic plasticity, adaptive responses, and ecological niche specialization (Figure 2).

### ML models for predicting *H. pylori* antimicrobial resistance phenotypes

**Figure 3.**
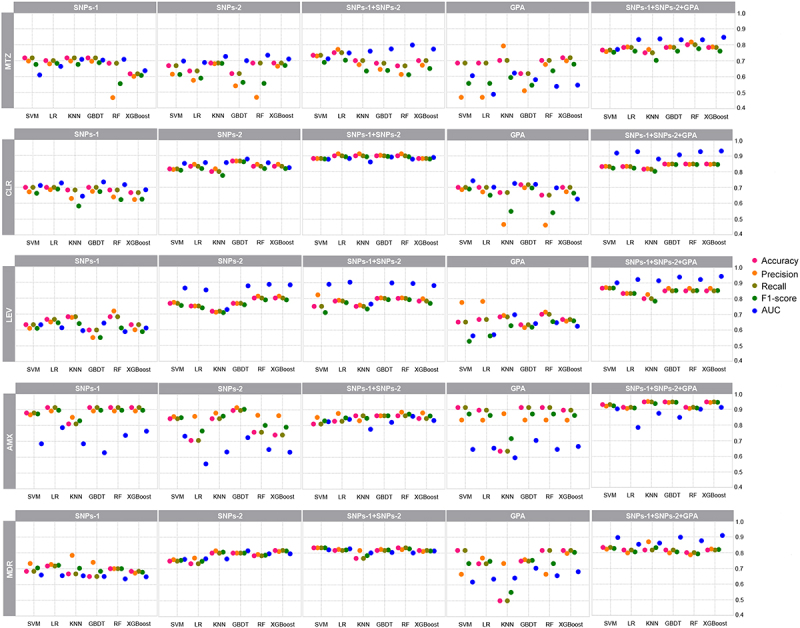
The predictive performances of six ML algorithms on the four antibiotics and MDR using different feature sets. Each individual panel displayed the performance of six ML algorithms in predicting five resistance phenotypes using five different feature sets. Abbreviations: SNP, single nucleotide polymorphisms; GPA, gene presence or absence; KNN, K-Nearest Neighbor; LR, Logistic Regression; SVM, Support Vector Machines; RF, Random Forest; GBDT, Gradient Boosting Decision Tree; XGBoost, eXtreme Gradient Boosting; MTZ, metronidazole; CLR, clarithromycin; LEV, levofloxacin; AMX, amoxicillin; MDR, multidrug-resistant.

Five different feature sets, SNPs-1, SNPs-2, SNPs-1+SNPs-2, GPA, and SNPs-1+SNPs-2+GPA, were employed. For each feature set, six ML models were constructed following feature selection using RF and LASSO methods. The predictive performance was then evaluated on the validation dataset using accuracy, precision, recall, F1-score, and AUC. The number of selected features for each feature set was shown in Supplementary Table S3, while the prediction metrics for the six ML models for each feature set are detailed in Figure 3 and Supplementary Table S4.

#### MTZ

**Figure 4.**
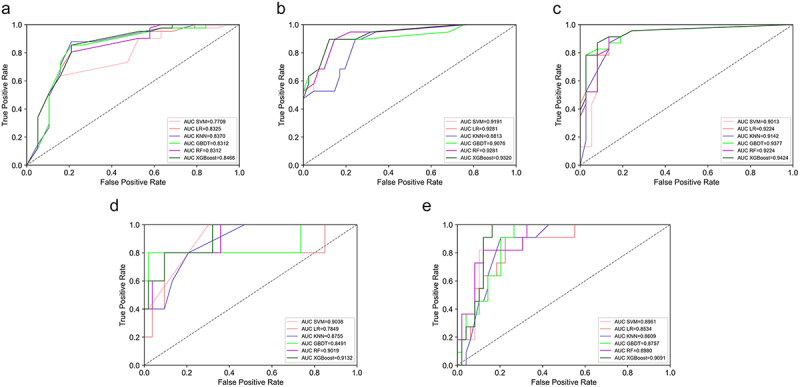
Comparison of ROC curves for antibiotic resistance prediction using different ML algorithms based on SNPs-1+SNPs-2+GPA in the validation set. (a-e) respectively presented the ROC curves for six ML algorithms applied to MTZ, CLR, LEV, AMX, and MDR based on the SNPs-1+SNPs-2+GPA feature set in the validation cohort. Abbreviations: AUC, the Area under the curve, KNN, K-Nearest Neighbor; LR, Logistic Regression; SVM, Support Vector Machines; RF, Random Forest; GBDT, Gradient Boosting Decision Tree; XGBoost, eXtreme Gradient Boosting.

First, we compared the performance of prediction models built solely on SNP sets. It was shown that using SNPs-1 set, KNN (AUC = 0.7080, Accuracy = 0.7167) provided more accurate predictions for MTZ resistance. When based on SNPs-2 set, RF exhibited better performance (AUC = 0.7330, Accuracy = 0.6833). However, the RF model for SNPs-1+SNPs-2 performed slightly better than the first two (AUC = 0.7978, Accuracy = 0.6667). On this basis, further integrating the GPA set showed that the SNPs-1+SNPs-2+GPA model outperformed those based on either SNPs-1+SNPs-2 or GPA sets alone. The XGBoost model demonstrated the best performance, with an AUC of 0.8466 (Figure 4(a)), an accuracy of 0.7833, a recall of 0.7833, a precision of 0.7863, and an F1-score of 0.7604.

#### CLR

Similarly, we constructed prediction models using the above approach. The results showed that the LR model performed best for the SNPs-1 set (AUC = 0.7272, Accuracy = 0.7000); the GBDT model performed best for the SNPs-2 set (AUC = 0.8793, Accuracy = 0.8667); and the GBDT model for SNPs-1+SNPs-2 outperformed the previous two (AUC = 0.8922, Accuracy = 0.9000). Further integrating the GPA set, the SNPs-1+SNPs-2+GPA model demonstrated superior performance compared to models based on either SNPs-1+SNPs-2 or GPA alone. The XGBoost model was the best, achieving an AUC of 0.9320 (Figure 4(b)), an accuracy and recall of 0.8500, a precision of 0.8474, and an F1-score of 0.8462.

#### LEV

All models based on the SNPs-1 set performed poorly, with AUCs below 0.65. The RF model for the SNPs-2 set performed better (AUC = 0.8866, Accuracy = 0.8000), and the LR model for the SNPs-1+SNPs-2 set showed improved performance (AUC = 0.9025, Accuracy = 0.7833). After integrating the GPA set, the SNPs-1+SNPs-2+GPA model outperformed those based on either SNPs-1+SNPs-2 or GPA alone. The XGBoost model was the best, with an AUC of 0.9424 (Figure 4(c)), an accuracy and recall of 0.8500, a precision of 0.8656, and an F1-score of 0.8519.

#### AMX

Similarly, the LR model performed best for the SNPs-1 set (AUC = 0.7868, Accuracy = 0.9138), while the SVM model was most effective for the SNPs-2 set (AUC = 0.7340, Accuracy = 0.8448). The RF model for SNPs-1+SNPs-2 showed improved performance (AUC = 0.8585, Accuracy = 0.8621). After integrating the GPA set, the SNPs-1+SNPs-2+GPA model outperformed the models based on GPA alone or SNPs-1+SNPs-2. The XGBoost model was the best, with an AUC of 0.9132 (Figure 4(d)), an accuracy and recall of 0.9483, a precision of 0.9446, and an F1-score of 0.9456.

#### MDR

Similarly, for models based solely on SNPs-1, the SVM model performed best (AUC = 0.6596, Accuracy = 0.6833). For SNPs-2, the GBDT model was most effective (AUC = 0.8145, Accuracy = 0.8000). The LR model for SNPs-1+SNPs-2 showed a slight improvement (AUC = 0.8265, Accuracy = 0.8167). The SNPs-1+SNPs-2+GPA model outperformed those based solely on GPA or SNPs-1+SNPs-2, with the XGBoost model achieving the best performance: AUC of 0.9091 (Figure 4e), accuracy and recall of 0.8167, precision of 0.8233, and an F1-score of 0.8197.

### SHAP interpretation of ML models

**Figure 5.**
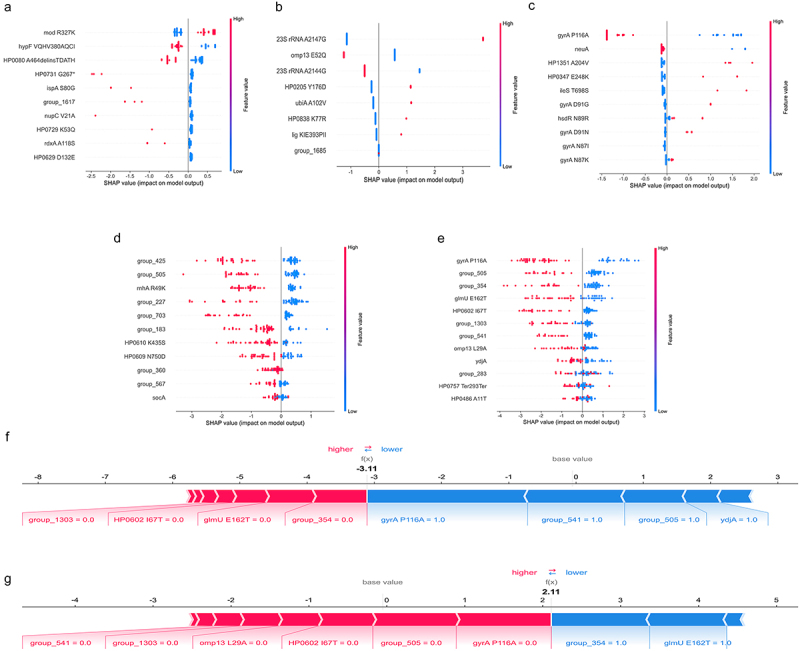
Global and local explanation of ML models using SHAP summary and force plots. (a-e) showed the overall interpretation of the XGBoost model using the SHAP summary plot for the predictions of MTZ, CLR, LEV, AMX, and MDR, respectively. Each dot represented a sample, and the redder the color, the larger the feature value, and the bluer the color, the lower the feature value. (f)provided an individualized local interpretation for one non-mdr strain; (g) provided an individualized local interpretation for one MDR strain. The bold numbers represented the predicted probabilities (f(x)), and the base value represented the average prediction. The length of the red or blue bars in the force plot was proportional to the contribution of the resistance features.

Subsequently, we employed SHAP values to perform interpretability analysis on the models for predicting resistance to the four antibiotics and MDR. This analysis aimed to reveal the features involved in the prediction models, along with their importance and contributions. Overall, the features associated with antibiotic resistance for each antibiotic can be categorized into three types, including previously identified in *H. pylori*, found in homologs of other species associated with antibiotic resistance or newly discovered (Supplementary Table S5). The 10 features of MTZ resistance prediction were linked to specific SNPs or GPAs. Among these, the R327K mutation in the *mod* gene stood out as a key indicator, demonstrating a positive association with MTZ resistance (Figure 5(a)). For CLR resistance prediction, the 23S rRNA A2147G and *omp13* E52Q mutations contributed the most to the model, followed by the 23S rRNA A2144G mutation (Figure 5(b)). For LEV resistance prediction, most of the red dots were clustered around the positive x-axis, indicating that the presence of SNP or gene increased *H. pylori* resistance to LEV (Figure 5(c)). It was noteworthy that known mutations associated with LEV resistance, such as *gyrA* D91 and N87, were also included in the list. The 11 features of AMX showed a negative association with resistance (Figure 5(d)). Among these, the top three features that contributed most significantly to AMX resistance prediction were *group_425*, *group_505*, and *rnhA* R49K. In terms of MDR prediction, the majority of the red dots were clustered around the negative x-axis, indicating that the absence of SNP or gene led to lower SHAP values, which meant a negative relevance with MDR (Figure 5(e)).

Next, we randomly selected sensitive or resistant isolates from the validation cohort to illustrate the individualized predictive ability of the model using SHAP force plots: one was a non-MDR strain and one was an MDR strain (Figure 5(f,g)). The arrows showed the influence of each factor on prediction. The blue and red arrows in the plot indicated whether the feature reduced or increased the likelihood of MDR. For the representative non-MDR strain, the SHAP value (−3.11) was lower than the base value (0); for the representative MDR strain, the SHAP value (2.11) was higher than the base value (0). These results demonstrated that the force plot offered a visual and intuitive interpretation of the contribution of each feature to the model’s prediction for MDR in individual *H. pylori* isolates.

### Model deployment

**Figure 6.**
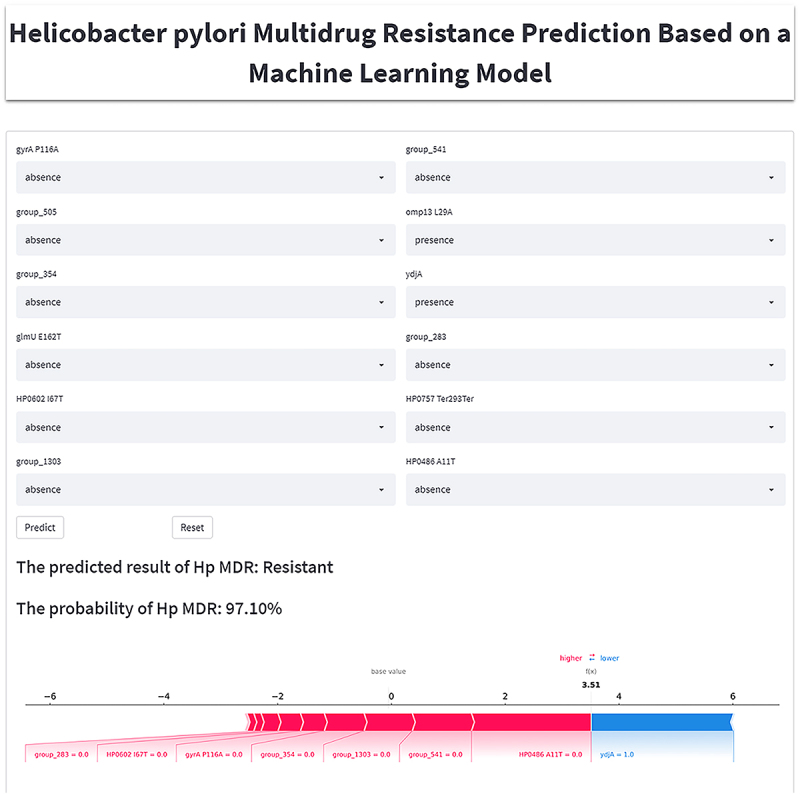
Schematic diagram of an online calculator used to predict the likelihood of developing MDR in patients with *H. pylori* infection.

**Figure 7.**
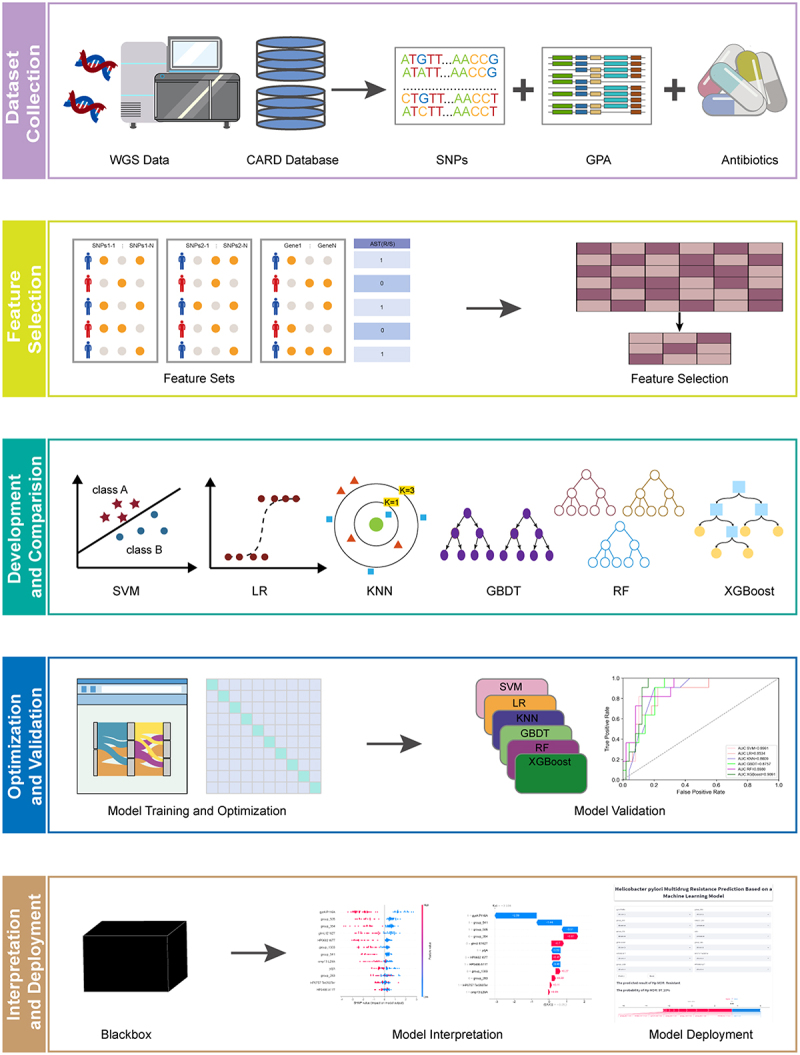
The workflow of using ML algorithms to predict antibiotic resistance in *H. pylori*.

Ultimately, we deployed a streamlined online application for predicting MDR in *H. pylori*. This application utilized the XGBoost model and offered accessibility to all physicians through the internet. Users could input the presence or absence of the 12 features required by the model, click “predict” to submit all parameters and the probabilities of MDR could be obtained, as shown in Figure 6. The workflow for developing the prediction models is shown in Figure 7.

## Discussion

In this study, we used whole-genome bacterial data and six different ML models, based on five feature sets to predict susceptibility to four common antibiotics and MDR in *H. pylori*. Next, we used SHAP to make the model outputs more intuitive, interpretable, and practical. And also, we developed a free, user-friendly web application for the optimal MDR model. This comprehensive approach not only enhances our understanding of the mechanisms of antibiotic resistance in *H. pylori* but also provides clinicians with practical tools to aid in clinical decision-making, ultimately contributing to more effective management of antibiotic resistance in *H. pylori*.

Currently, the techniques used for detecting antibiotic resistance in *H. pylori* primarily rely on culture-based and PCR-based methods. These tests are time-consuming and lake insight into the genetic basis of resistance or identification of potential resistance mechanisms. In contrast, WGS data provides comprehensive information on all genes, allowing the identification of both known and novel antibiotic resistance-related genes. In this study, we downloaded the WGS data of 296 strains with antibiotic resistance information from the BV-BRC and NCBI databases. Utilizing pan-genome analysis and SNP calling, we obtained comprehensive genetic variation data, including both GPA and SNPs-1. Subsequently, we identified 91 known antibiotic resistance-related SNPs from the CARD database (SNPs-2). When compared the performance of predictive models from different SNP sets, we found that, except for AMX, the models based on the SNPs-2 significantly outperformed those based on the SNPs-1. Integrating SNPs-1 and SNPs-2 yielded even better performance for all kinds of antibiotics. Models based solely on SNPs-1, derived from whole-genome SNP calling, performed less effectively, likely due to the inclusion of numerous variants unrelated to antibiotic resistance. While the CARD database provides information on known antibiotic resistance SNPs, it may not cover all resistance-related genetic variations, leading to suboptimal model performance. The superior performance of the integrated SNPs-1+SNPs-2 model suggested that combining these sets offered more comprehensive coverage of both known and unknown resistance-related SNPs, thereby enhancing the accuracy of antibiotic resistance predictions.

While mutations are known to be a primary cause of *H. pylori* resistance, antibiotic resistance can also be influenced by the presence or absence of specific genes. These genes may directly confer or enhance resistance, or affect bacterial survival by regulating gene expression or providing alternative metabolic pathways. Therefore, we integrated the GPA set with SNPs-1 and SNPs-2 sets, and the results showed that this combined model demonstrated superior predictive ability for antimicrobial resistance classification compared to other feature sets. This suggests that incorporating pan-genome analysis data has a positive impact on antibiotic resistance prediction. Our findings indicated that using ML techniques to integrate genomic and resistance data effectively predicts antimicrobial resistance in *H. pylori*. Moreover, this multi-layered data integration strategy significantly improves the overall predictive performance of the models.

Interpreting the decision-making process of ML algorithms is pivotal when applying these methods, especially in genotype–phenotype prediction for AST. Therefore, we visualized the best ML models for each antibiotic by SHAP, providing global and local explanations for these “black box” models. The global explanation involved ranking and visualization of the features in models. Local explanations showed individual reasons for antibiotic resistance and quantified feature contributions. This detailed view allowed for the identification of specific resistance mechanisms in *H. pylori* and supported personalized eradication strategies. All resistance features could be categorized into three groups: previously identified resistance features in *H. pylori*, newly identified features, and features found in homologous genes associated with antibiotic resistance in other species. Among the features previously identified in *H. pylori* were mutations such as A2144G and A2147G mutations in the 23S rRNA gene associated with CLR resistance [21,22], D91 and N87 mutations in the *gyrA* gene associated with LEV resistance [23] and mutations in the *rdxA* gene associated with MTZ resistance [24]. We also identified a number of features found in homologous genes associated with antibiotic resistance in other species, including *neuA* [25], *glmU* [26], *ileS* [27], *hsdR* [28], *ubiA* [29], *nupC* [30], *rnhA* [31], *ispA* [32], *lig* [33], *hypF* [34], *mod* [35], *ydjA* [36], and *omp13* [37,38]. This class of genes offers opportunities to further understand and address *H. pylori* antibiotic resistance. They may affect bacterial drug resistance through a variety of mechanisms, such as altering cell wall structure, metabolic pathways, and protein function. By further exploring the roles and mechanisms of these genes in *H. pylori*, we may develop new antimicrobial drug targets or design novel combination therapies to address existing antibiotic resistance issues. In addition, we discovered some new potential resistance genes with unknown functions, such as *group_354*, *group_505* and *group_1303*. Although the specific functions of these genes are not yet clear, they provide valuable directions for future research. Through further functional validation and mechanistic studies, we expect to reveal the specific roles of these genes in drug resistance and assess their potential as novel antimicrobial drug targets.

Moreover, to make the results of ML models more easily communicable and understandable, enhancing their practicality and applicability, we developed a web-based application featuring network calculator for the top-performing XGBoost model in MDR predicting. This application will assist physicians in identifying whether the *H. pylori* is an MDR strain and visualizing, through SHAP force plots, why the model makes this prediction.

This study has several notable strengths. It pioneers the integration of WGS data and six ML models for predicting *H. pylori* antibiotic resistance. WGS provides a thorough examination of the entire genome, enabling the identification of both known and novel antibiotic resistance genes, which is a significant advantage over traditional methods that are limited to detecting known resistance genes. The genes and mutations screened by our method have the potential to be integrated into existing antimicrobial resistance databases, improving their comprehensiveness and usefulness. Additionally, we successfully applied SHAP values to explain the “black box” of ML. Global explanations ranked the features predicting resistance, while local explanations clarified individual resistance mechanisms. Moreover, to make the model more practical, we developed a web-based application to inform clinicians about the probability of *H. pylori* developing MDR to commonly used antibiotics. We anticipate that this application will advance the development of antibiotic resistance detection tools for *H. pylori* and reveal new resistance mechanisms.

Indeed, this study has some limitations. For one, although we have made efforts to retrieve all available *H. pylori* WGS data and associated drug resistance information from public databases, the number of samples available for analysis remains limited, and lacks further external validation. Future research should aim to expand the dataset by including more samples from diverse geographical regions and different clinical settings. In addition, bacterial plasmids play a crucial role in the spread of antibiotic resistance. Plasmids carrying multiple resistance genes contribute to antibiotic resistance being a global public health challenge. However, among the genomic data included in this study, only four cases contained plasmid information, and plasmid genes could not be incorporated into the model construction. Future efforts should focus on collecting and integrating genomic data that includes plasmid information to enhance the comprehensiveness and accuracy of the models. Furthermore, the web interface currently requires users to input the mutations or genes to get the antimicrobial resistance information. The user experience would be significantly enhanced if the application could automatically identify the variables required for analysis when uploading a genome. However, due to the complexity and computational demands in web applications, we are currently unable to implement this feature, which may cause difficulties for clinicians. In the future, we are committed to enhancing user-friendliness. Finally, integrating MIC data with ML models could improve the assessment of the relationship between genes and resistance. However, in our dataset, the number of strains with explicit MIC values was 121 for CLR, 123 for LEV, and 115 for AMX, while only 39 strains had MIC values for MTZ. This limitation hampers the effective integration of MIC data into the ML models. Future work should focus on increasing the number of strains with defined MIC values to address this gap and enhance the robustness of the models.

## Conclusions

Our study demonstrates the potential of leveraging WGS data alongside ML algorithms to predict antibiotic resistance in *H. pylori*. By incorporating SHAP, we improve the interpretability of the ML models and enhance the understanding of resistance features. The features identified through SHAP will not only steer the direction of future research endeavors but also serve as potential targets for the development of innovative diagnostic tools and therapeutic interventions tailored to *H. pylori*. We anticipate that personalized eradication therapy for *H. pylori* will reduce the empirical use of broad-spectrum antibiotics in clinical practice, potentially lowering treatment costs, improving eradication rates, and ultimately leading to better clinical outcomes for infection-related diseases.

## Supplementary Material

Supplementary Table S5.docx

Supplementary Table S1.xlsx

Supplementary Table S4.docx

Supplementary Table S3.xlsx

Supplementary Figure S1.tif

Supplementary Table S2.docx

## Data Availability

The data generated during the study, including raw data and supplementary materials (Dataset EV1), are available at figshare at http://doi.org/10.6084/m9.figshare.26518687. The optimal model and Python code are archived on Zenodo (DOI: 10.5281/zenodo.15007638) and accessible via GitHub (https://github.com/H.pylori/MDR), both under an MIT open-source licence.

## Permission to Use Datasets

The whole-genome sequencing data used in this study were obtained from publicly accessible databases, including NCBI (https://www.ncbi.nlm.nih.gov/) and BV-BRC (https://www.bv-brc.org/). These datasets are available under open licences that permit unrestricted reuse for research purposes. Therefore, no additional permissions were needed to access or use these data.
