# Supplementary material for: Assessment for antibiotic resistance in Helicobacter pylori: A practical and interpretable machine learning model based on genome-wide genetic variation
Source: Virulence. 2025 Mar 21;16(1):2481503. doi: 10.1080/21505594.2025.2481503 (PMC11934168; doi:10.1080/21505594.2025.2481503)
Supplement: Supplementary Table S5.docx [file KVIR_A_2481503_SM2211.docx]

**Supplementary Table S5. The features associated with antibiotic resistance for each antibiotic revealed by SHAP**

| **Antibiotics** | **Features** | **Previously identified in *H. pylori*** | **Found in homologs of other species** | **Newly discovered** |
| --- | --- | --- | --- | --- |
| **MTZ** | *mod* R327K |  | √ |  |
| **MTZ** | *hypF* VQHV380AQCI |  | √ |  |
| **MTZ** | *HP0080* A464delinsTDATH |  |  | √ |
| **MTZ** | *HP0731* G267* |  |  | √ |
| **MTZ** | *ispA* S80G |  | √ |  |
| **MTZ** | *group_1617* |  |  | √ |
| **MTZ** | *nupC* V21A |  | √ |  |
| **MTZ** | *HP0729* K53Q |  |  | √ |
| **MTZ** | *rdxA* A118S | √ |  |  |
| **MTZ** | *HP0629* D132E |  |  | √ |
| **CLR** | 23S rRNA A2147G | √ |  |  |
| **CLR** | *omp13* E52Q |  | √ |  |
| **CLR** | 23S rRNA A2144G | √ |  |  |
| **CLR** | *HP0205* Y176D |  |  | √ |
| **CLR** | *ubiA* A102V |  | √ |  |
| **CLR** | *HP0838* K77R |  |  | √ |
| **CLR** | *lig* KIE393PII |  | √ |  |
| **CLR** | *group_1685* |  |  | √ |
| **LEV** | *gyrA* P116A |  | √ |  |
| **LEV** | *neuA* |  | √ |  |
| **LEV** | *HP1351* A204V |  |  | √ |
| **LEV** | *HP0347* E248K |  |  | √ |
| **LEV** | *ileS* T698S |  | √ |  |
| **LEV** | *gyrA* D91G | √ |  |  |
| **LEV** | *hsdR* N89R |  | √ |  |
| **LEV** | *gyrA* D91N | √ |  |  |
| **LEV** | *gyrA* N87I | √ |  |  |
| **LEV** | *gyrA* N87K | √ |  |  |
| **AMX** | *group_425* |  |  | √ |
| **AMX** | *group_505* |  |  | √ |
| **AMX** | *rnhA* R49K |  | √ |  |
| **AMX** | *group_227* |  |  | √ |
| **AMX** | *group_703* |  |  | √ |
| **AMX** | *group_183* |  |  | √ |
| **AMX** | *HP0610* K435S |  |  | √ |
| **AMX** | *HP0609* N750D |  |  | √ |
| **AMX** | *group_360* |  |  | √ |
| **AMX** | *group_567* |  |  | √ |
| **AMX** | *socA* |  |  | √ |
| **MDR** | *gyrA* P116A |  | √ |  |
| **MDR** | *group_505* |  |  | √ |
| **MDR** | *group_354* |  |  | √ |
| **MDR** | *glmU* E162T |  | √ |  |
| **MDR** | *HP0602* I67T |  |  | √ |
| **MDR** | *group_1303* |  |  | √ |
| **MDR** | *group_541* |  |  | √ |
| **MDR** | *omp13* L29A |  | √ |  |
| **MDR** | *ydjA* |  | √ |  |
| **MDR** | *group_283* |  |  | √ |
| **MDR** | *HP0757* Ter293Ter |  |  | √ |
| **MDR** | *HP0486* A11T |  |  | √ |

Abbreviations: metronidazole, MTZ; clarithromycin, CLR; levofloxacin, LEV; amoxicillin, AMX; multidrug-resistant, MDR.
