# Supplementary material for: Assessment for antibiotic resistance in Helicobacter pylori: A practical and interpretable machine learning model based on genome-wide genetic variation
Source: Virulence. 2025 Mar 21;16(1):2481503. doi: 10.1080/21505594.2025.2481503 (PMC11934168; doi:10.1080/21505594.2025.2481503)
Supplement: Supplementary Table S4.docx [file KVIR_A_2481503_SM2209.docx]

**Supplementary Table S4. Comparison of the predictive performance of six ML models in the validation cohort**

| **Antibiotics** | **ML models** | **Accuracy** | **Precision** | **Recall** | **F1-score** | **AUC** | **Feature sets** |
| --- | --- | --- | --- | --- | --- | --- | --- |
| **MTZ** | SVM | 0.7167 | 0.6973 | 0.7167 | 0.6757 | 0.6091 | SNPs-1 |
| **MTZ** | LR | 0.7000 | 0.6794 | 0.7000 | 0.6821 | 0.6630 | SNPs-1 |
| **MTZ** | KNN | 0.7167 | 0.6973 | 0.7167 | 0.6757 | 0.7080 | SNPs-1 |
| **MTZ** | GBDT | 0.7167 | 0.6957 | 0.7167 | 0.6867 | 0.7028 | SNPs-1 |
| **MTZ** | RF | 0.6833 | 0.4669 | 0.6833 | 0.5548 | 0.7080 | SNPs-1 |
| **MTZ** | XGBoost | 0.6167 | 0.6002 | 0.6167 | 0.6070 | 0.6361 | SNPs-1 |
| **MTZ** | SVM | 0.6667 | 0.6128 | 0.6667 | 0.6110 | 0.6958 | SNPs-2 |
| **MTZ** | LR | 0.6333 | 0.5745 | 0.6333 | 0.5878 | 0.6868 | SNPs-2 |
| **MTZ** | KNN | 0.6833 | 0.6790 | 0.6833 | 0.6810 | 0.7246 | SNPs-2 |
| **MTZ** | GBDT | 0.6167 | 0.5391 | 0.6167 | 0.5613 | 0.6983 | SNPs-2 |
| **MTZ** | RF | 0.6833 | 0.4669 | 0.6833 | 0.5548 | 0.7330 | SNPs-2 |
| **MTZ** | XGBoost | 0.6833 | 0.6634 | 0.6833 | 0.6684 | 0.7086 | SNPs-2 |
| **MTZ** | SVM | 0.7333 | 0.7290 | 0.7333 | 0.6888 | 0.7105 | SNPs-1+SNPs-2 |
| **MTZ** | LR | 0.7500 | 0.7701 | 0.7500 | 0.7021 | 0.7478 | SNPs-1+SNPs-2 |
| **MTZ** | KNN | 0.7000 | 0.6745 | 0.7000 | 0.6344 | 0.7593 | SNPs-1+SNPs-2 |
| **MTZ** | GBDT | 0.6833 | 0.6446 | 0.6833 | 0.6376 | 0.7734 | SNPs-1+SNPs-2 |
| **MTZ** | RF | 0.6667 | 0.6128 | 0.6667 | 0.6110 | 0.7978 | SNPs-1+SNPs-2 |
| **MTZ** | XGBoost | 0.7000 | 0.6709 | 0.7000 | 0.6499 | 0.7721 | SNPs-1+SNPs-2 |
| **MTZ** | SVM | 0.6833 | 0.4669 | 0.6833 | 0.5548 | 0.6033 | GPA |
| **MTZ** | LR | 0.6833 | 0.4669 | 0.6833 | 0.5548 | 0.4859 | GPA |
| **MTZ** | KNN | 0.7000 | 0.7915 | 0.7000 | 0.5920 | 0.6200 | GPA |
| **MTZ** | GBDT | 0.6167 | 0.5083 | 0.6167 | 0.5432 | 0.5783 | GPA |
| **MTZ** | RF | 0.7000 | 0.6745 | 0.7000 | 0.6344 | 0.5359 | GPA |
| **MTZ** | XGBoost | 0.7167 | 0.6973 | 0.7167 | 0.6757 | 0.5436 | GPA |
| **MTZ** | SVM | 0.7667 | 0.7572 | 0.7667 | 0.7527 | 0.7709 | SNPs-1+SNPs-2+GPA |
| **MTZ** | LR | 0.7833 | 0.7863 | 0.7833 | 0.7604 | 0.8325 | SNPs-1+SNPs-2+GPA |
| **MTZ** | KNN | 0.7500 | 0.7701 | 0.7500 | 0.7021 | 0.8370 | SNPs-1+SNPs-2+GPA |
| **MTZ** | GBDT | 0.7833 | 0.7863 | 0.7833 | 0.7604 | 0.8312 | SNPs-1+SNPs-2+GPA |
| **MTZ** | RF | 0.8000 | 0.8174 | 0.8000 | 0.7752 | 0.8312 | SNPs-1+SNPs-2+GPA |
| **MTZ** | XGBoost | 0.7833 | 0.7863 | 0.7833 | 0.7604 | 0.8466 | SNPs-1+SNPs-2+GPA |
| **CLR** | SVM | 0.7000 | 0.6717 | 0.7000 | 0.6627 | 0.7118 | SNPs-1 |
| **CLR** | LR | 0.7000 | 0.6852 | 0.7000 | 0.6893 | 0.7272 | SNPs-1 |
| **CLR** | KNN | 0.6833 | 0.6296 | 0.6833 | 0.5823 | 0.6444 | SNPs-1 |
| **CLR** | GBDT | 0.7000 | 0.6748 | 0.7000 | 0.6733 | 0.7336 | SNPs-1 |
| **CLR** | RF | 0.6833 | 0.6392 | 0.6833 | 0.6227 | 0.7169 | SNPs-1 |
| **CLR** | XGBoost | 0.6667 | 0.6231 | 0.6667 | 0.6253 | 0.6849 | SNPs-1 |
| **CLR** | SVM | 0.8167 | 0.8133 | 0.8167 | 0.8080 | 0.8517 | SNPs-2 |
| **CLR** | LR | 0.8333 | 0.8457 | 0.8333 | 0.8185 | 0.8569 | SNPs-2 |
| **CLR** | KNN | 0.8000 | 0.8174 | 0.8000 | 0.7752 | 0.8569 | SNPs-2 |
| **CLR** | GBDT | 0.8667 | 0.8667 | 0.8667 | 0.8619 | 0.8793 | SNPs-2 |
| **CLR** | RF | 0.8333 | 0.8457 | 0.8333 | 0.8185 | 0.8556 | SNPs-2 |
| **CLR** | XGBoost | 0.8333 | 0.8457 | 0.8333 | 0.8185 | 0.8248 | SNPs-2 |
| **CLR** | SVM | 0.8833 | 0.8828 | 0.8833 | 0.8804 | 0.8787 | SNPs-1+SNPs-2 |
| **CLR** | LR | 0.9000 | 0.9128 | 0.9000 | 0.8940 | 0.8832 | SNPs-1+SNPs-2 |
| **CLR** | KNN | 0.9000 | 0.9128 | 0.9000 | 0.8940 | 0.8614 | SNPs-1+SNPs-2 |
| **CLR** | GBDT | 0.9000 | 0.9030 | 0.9000 | 0.8964 | 0.8922 | SNPs-1+SNPs-2 |
| **CLR** | RF | 0.9000 | 0.9128 | 0.9000 | 0.8940 | 0.8793 | SNPs-1+SNPs-2 |
| **CLR** | XGBoost | 0.8833 | 0.8828 | 0.8833 | 0.8804 | 0.8896 | SNPs-1+SNPs-2 |
| **CLR** | SVM | 0.7000 | 0.6852 | 0.7000 | 0.6893 | 0.7420 | GPA |
| **CLR** | LR | 0.7000 | 0.6709 | 0.7000 | 0.6499 | 0.7009 | GPA |
| **CLR** | KNN | 0.6667 | 0.4633 | 0.6667 | 0.5467 | 0.7246 | GPA |
| **CLR** | GBDT | 0.7167 | 0.6972 | 0.7167 | 0.6958 | 0.7189 | GPA |
| **CLR** | RF | 0.6500 | 0.4595 | 0.6500 | 0.5384 | 0.6970 | GPA |
| **CLR** | XGBoost | 0.7000 | 0.6717 | 0.7000 | 0.6627 | 0.6252 | GPA |
| **CLR** | SVM | 0.8333 | 0.8350 | 0.8333 | 0.8234 | 0.9191 | SNPs-1+SNPs-2+GPA |
| **CLR** | LR | 0.8333 | 0.8350 | 0.8333 | 0.8234 | 0.9281 | SNPs-1+SNPs-2+GPA |
| **CLR** | KNN | 0.8167 | 0.8191 | 0.8167 | 0.8032 | 0.8813 | SNPs-1+SNPs-2+GPA |
| **CLR** | GBDT | 0.8500 | 0.8474 | 0.8500 | 0.8462 | 0.9076 | SNPs-1+SNPs-2+GPA |
| **CLR** | RF | 0.8500 | 0.8474 | 0.8500 | 0.8462 | 0.9281 | SNPs-1+SNPs-2+GPA |
| **CLR** | XGBoost | 0.8500 | 0.8474 | 0.8500 | 0.8462 | 0.9320 | SNPs-1+SNPs-2+GPA |
| **LEV** | SVM | 0.6333 | 0.6101 | 0.6333 | 0.6101 | 0.6328 | SNPs-1 |
| **LEV** | LR | 0.6667 | 0.6501 | 0.6667 | 0.6455 | 0.6136 | SNPs-1 |
| **LEV** | KNN | 0.6833 | 0.6791 | 0.6833 | 0.6401 | 0.5957 | SNPs-1 |
| **LEV** | GBDT | 0.6000 | 0.5520 | 0.6000 | 0.5523 | 0.6435 | SNPs-1 |
| **LEV** | RF | 0.6833 | 0.7194 | 0.6833 | 0.6126 | 0.5897 | SNPs-1 |
| **LEV** | XGBoost | 0.6333 | 0.6013 | 0.6333 | 0.5896 | 0.6124 | SNPs-1 |
| **LEV** | SVM | 0.7667 | 0.7726 | 0.7667 | 0.7535 | 0.8631 | SNPs-2 |
| **LEV** | LR | 0.7500 | 0.7500 | 0.7500 | 0.7384 | 0.8514 | SNPs-2 |
| **LEV** | KNN | 0.7167 | 0.7107 | 0.7167 | 0.7084 | 0.7280 | SNPs-2 |
| **LEV** | GBDT | 0.7667 | 0.7664 | 0.7667 | 0.7579 | 0.8772 | SNPs-2 |
| **LEV** | RF | 0.8000 | 0.8119 | 0.8000 | 0.7887 | 0.8866 | SNPs-2 |
| **LEV** | XGBoost | 0.8000 | 0.8119 | 0.8000 | 0.7887 | 0.8831 | SNPs-2 |
| **LEV** | SVM | 0.7500 | 0.8221 | 0.7500 | 0.7106 | 0.8895 | SNPs-1+SNPs-2 |
| **LEV** | LR | 0.7833 | 0.7880 | 0.7833 | 0.7733 | 0.9025 | SNPs-1+SNPs-2 |
| **LEV** | KNN | 0.7500 | 0.7570 | 0.7500 | 0.7331 | 0.7644 | SNPs-1+SNPs-2 |
| **LEV** | GBDT | 0.8000 | 0.8033 | 0.8000 | 0.7925 | 0.8978 | SNPs-1+SNPs-2 |
| **LEV** | RF | 0.8000 | 0.8033 | 0.8000 | 0.7925 | 0.8942 | SNPs-1+SNPs-2 |
| **LEV** | XGBoost | 0.7833 | 0.7978 | 0.7833 | 0.7687 | 0.8813 | SNPs-1+SNPs-2 |
| **LEV** | SVM | 0.6500 | 0.7746 | 0.6500 | 0.5281 | 0.5628 | GPA |
| **LEV** | LR | 0.6667 | 0.7816 | 0.6667 | 0.5625 | 0.5700 | GPA |
| **LEV** | KNN | 0.6833 | 0.6921 | 0.6833 | 0.6275 | 0.6968 | GPA |
| **LEV** | GBDT | 0.6333 | 0.6152 | 0.6333 | 0.6178 | 0.6406 | GPA |
| **LEV** | RF | 0.7000 | 0.7135 | 0.7000 | 0.6533 | 0.6465 | GPA |
| **LEV** | XGBoost | 0.6667 | 0.6561 | 0.6667 | 0.6583 | 0.6238 | GPA |
| **LEV** | SVM | 0.8667 | 0.8705 | 0.8667 | 0.8676 | 0.9013 | SNPs-1+SNPs-2+GPA |
| **LEV** | LR | 0.8333 | 0.8333 | 0.8333 | 0.8333 | 0.9224 | SNPs-1+SNPs-2+GPA |
| **LEV** | KNN | 0.8000 | 0.8262 | 0.8000 | 0.7841 | 0.9142 | SNPs-1+SNPs-2+GPA |
| **LEV** | GBDT | 0.8500 | 0.8656 | 0.8500 | 0.8519 | 0.9377 | SNPs-1+SNPs-2+GPA |
| **LEV** | RF | 0.8500 | 0.8656 | 0.8500 | 0.8519 | 0.9224 | SNPs-1+SNPs-2+GPA |
| **LEV** | XGBoost | 0.8500 | 0.8656 | 0.8500 | 0.8519 | 0.9424 | SNPs-1+SNPs-2+GPA |
| **AMX** | SVM | 0.8793 | 0.8677 | 0.8793 | 0.8732 | 0.6849 | SNPs-1 |
| **AMX** | LR | 0.9138 | 0.8916 | 0.9138 | 0.8965 | 0.7868 | SNPs-1 |
| **AMX** | KNN | 0.8103 | 0.8515 | 0.8103 | 0.8295 | 0.6849 | SNPs-1 |
| **AMX** | RF | 0.9138 | 0.8916 | 0.9138 | 0.8965 | 0.6283 | SNPs-1 |
| **AMX** | GBDT | 0.9138 | 0.8916 | 0.9138 | 0.8965 | 0.7377 | SNPs-1 |
| **AMX** | XGBoost | 0.9138 | 0.8916 | 0.9138 | 0.8965 | 0.7642 | SNPs-1 |
| **AMX** | SVM | 0.8448 | 0.8579 | 0.8448 | 0.8511 | 0.7340 | SNPs-2 |
| **AMX** | LR | 0.7069 | 0.8593 | 0.7069 | 0.7667 | 0.5585 | SNPs-2 |
| **AMX** | KNN | 0.8448 | 0.8805 | 0.8448 | 0.8605 | 0.6340 | SNPs-2 |
| **AMX** | GBDT | 0.8966 | 0.9149 | 0.8966 | 0.9042 | 0.7245 | SNPs-2 |
| **AMX** | RF | 0.7586 | 0.8661 | 0.7586 | 0.8024 | 0.6491 | SNPs-2 |
| **AMX** | XGBoost | 0.7414 | 0.8638 | 0.7414 | 0.7906 | 0.6321 | SNPs-2 |
| **AMX** | SVM | 0.8103 | 0.8515 | 0.8103 | 0.8295 | 0.8245 | SNPs-1+SNPs-2 |
| **AMX** | LR | 0.8276 | 0.8770 | 0.8276 | 0.8488 | 0.8396 | SNPs-1+SNPs-2 |
| **AMX** | KNN | 0.8621 | 0.8307 | 0.8621 | 0.8461 | 0.7774 | SNPs-1+SNPs-2 |
| **AMX** | GBDT | 0.8621 | 0.8621 | 0.8621 | 0.8621 | 0.8208 | SNPs-1+SNPs-2 |
| **AMX** | RF | 0.8621 | 0.8847 | 0.8621 | 0.8722 | 0.8585 | SNPs-1+SNPs-2 |
| **AMX** | XGBoost | 0.8448 | 0.8805 | 0.8448 | 0.8605 | 0.8321 | SNPs-1+SNPs-2 |
| **AMX** | SVM | 0.9138 | 0.8350 | 0.9138 | 0.8726 | 0.6491 | GPA |
| **AMX** | LR | 0.8966 | 0.8336 | 0.8966 | 0.8639 | 0.6566 | GPA |
| **AMX** | KNN | 0.6379 | 0.8748 | 0.6379 | 0.7173 | 0.5962 | GPA |
| **AMX** | GBDT | 0.9138 | 0.8350 | 0.9138 | 0.8726 | 0.7057 | GPA |
| **AMX** | RF | 0.9138 | 0.8350 | 0.9138 | 0.8726 | 0.6491 | GPA |
| **AMX** | XGBoost | 0.8966 | 0.8336 | 0.8966 | 0.8639 | 0.6679 | GPA |
| **AMX** | SVM | 0.9310 | 0.9214 | 0.9310 | 0.9231 | 0.9038 | SNPs-1+SNPs-2+GPA |
| **AMX** | LR | 0.9138 | 0.9061 | 0.9138 | 0.9094 | 0.7849 | SNPs-1+SNPs-2+GPA |
| **AMX** | KNN | 0.9483 | 0.9510 | 0.9483 | 0.9379 | 0.8755 | SNPs-1+SNPs-2+GPA |
| **AMX** | GBDT | 0.9483 | 0.9446 | 0.9483 | 0.9456 | 0.8491 | SNPs-1+SNPs-2+GPA |
| **AMX** | RF | 0.9138 | 0.9061 | 0.9138 | 0.9094 | 0.9019 | SNPs-1+SNPs-2+GPA |
| **AMX** | XGBoost | 0.9483 | 0.9446 | 0.9483 | 0.9456 | 0.9132 | SNPs-1+SNPs-2+GPA |
| **MDR** | SVM | 0.6833 | 0.7326 | 0.6833 | 0.7041 | 0.6596 | SNPs-1 |
| **MDR** | LR | 0.7167 | 0.7264 | 0.7167 | 0.7214 | 0.6558 | SNPs-1 |
| **MDR** | KNN | 0.6667 | 0.7842 | 0.6667 | 0.7022 | 0.6549 | SNPs-1 |
| **MDR** | GBDT | 0.6500 | 0.7400 | 0.6500 | 0.6831 | 0.6503 | SNPs-1 |
| **MDR** | RF | 0.7000 | 0.7000 | 0.7000 | 0.7000 | 0.6354 | SNPs-1 |
| **MDR** | XGBoost | 0.6833 | 0.6717 | 0.6833 | 0.6774 | 0.6484 | SNPs-1 |
| **MDR** | SVM | 0.7500 | 0.7587 | 0.7500 | 0.7541 | 0.7607 | SNPs-2 |
| **MDR** | LR | 0.7333 | 0.7689 | 0.7333 | 0.7482 | 0.7644 | SNPs-2 |
| **MDR** | KNN | 0.8000 | 0.8144 | 0.8000 | 0.8063 | 0.7635 | SNPs-2 |
| **MDR** | GBDT | 0.8000 | 0.8000 | 0.8000 | 0.8000 | 0.8145 | SNPs-2 |
| **MDR** | RF | 0.7833 | 0.7910 | 0.7833 | 0.7869 | 0.7959 | SNPs-2 |
| **MDR** | XGBoost | 0.8167 | 0.8103 | 0.8167 | 0.8132 | 0.7959 | SNPs-2 |
| **MDR** | SVM | 0.8333 | 0.8333 | 0.8333 | 0.8333 | 0.8210 | SNPs-1+SNPs-2 |
| **MDR** | LR | 0.8167 | 0.8233 | 0.8167 | 0.8197 | 0.8265 | SNPs-1+SNPs-2 |
| **MDR** | KNN | 0.7667 | 0.8162 | 0.7667 | 0.7841 | 0.8015 | SNPs-1+SNPs-2 |
| **MDR** | GBDT | 0.8167 | 0.8233 | 0.8167 | 0.8197 | 0.8043 | SNPs-1+SNPs-2 |
| **MDR** | RF | 0.8333 | 0.8224 | 0.8333 | 0.8267 | 0.8015 | SNPs-1+SNPs-2 |
| **MDR** | XGBoost | 0.8167 | 0.8103 | 0.8167 | 0.8132 | 0.8135 | SNPs-1+SNPs-2 |
| **MDR** | SVM | 0.8167 | 0.6669 | 0.8167 | 0.7343 | 0.6178 | GPA |
| **MDR** | LR | 0.7333 | 0.7689 | 0.7333 | 0.7482 | 0.6364 | GPA |
| **MDR** | KNN | 0.5000 | 0.7346 | 0.5000 | 0.5526 | 0.6429 | GPA |
| **MDR** | GBDT | 0.7500 | 0.7587 | 0.7500 | 0.7541 | 0.7050 | GPA |
| **MDR** | RF | 0.8167 | 0.6669 | 0.8167 | 0.7343 | 0.6577 | GPA |
| **MDR** | XGBoost | 0.8167 | 0.7984 | 0.8167 | 0.8049 | 0.6827 | GPA |
| **MDR** | SVM | 0.8333 | 0.8224 | 0.8333 | 0.8267 | 0.8961 | SNPs-1+SNPs-2+GPA |
| **MDR** | LR | 0.8167 | 0.7984 | 0.8167 | 0.8049 | 0.8534 | SNPs-1+SNPs-2+GPA |
| **MDR** | KNN | 0.8167 | 0.8694 | 0.8167 | 0.8317 | 0.8609 | SNPs-1+SNPs-2+GPA |
| **MDR** | RF | 0.8167 | 0.7984 | 0.8167 | 0.8049 | 0.8980 | SNPs-1+SNPs-2+GPA |
| **MDR** | GBDT | 0.8000 | 0.7861 | 0.8000 | 0.7920 | 0.8757 | SNPs-1+SNPs-2+GPA |
| **MDR** | XGBoost | 0.8167 | 0.8233 | 0.8167 | 0.8197 | 0.9091 | SNPs-1+SNPs-2+GPA |

Abbreviations: MTZ, metronidazole; CLR, clarithromycin; LEV, levofloxacin; AMX, amoxicillin; MDR, multidrug-resistant; ML, machine learning; AUC, the Area Under the Curve; SNP, single nucleotide polymorphisms; GPA, gene presence or absence; KNN, K-Nearest Neighbor; LR, Logistic Regression; SVM, Support Vector Machines; RF, Random Forest; GBDT, Gradient Boosting Decision Tree; XGBoost, eXtreme Gradient Boosting.
