# Supplementary figures and images for: Assessment for antibiotic resistance in Helicobacter pylori: A practical and interpretable machine learning model based on genome-wide genetic variation
Source: Virulence. 2025 Mar 21;16(1):2481503. doi: 10.1080/21505594.2025.2481503 (PMC11934168; doi:10.1080/21505594.2025.2481503)

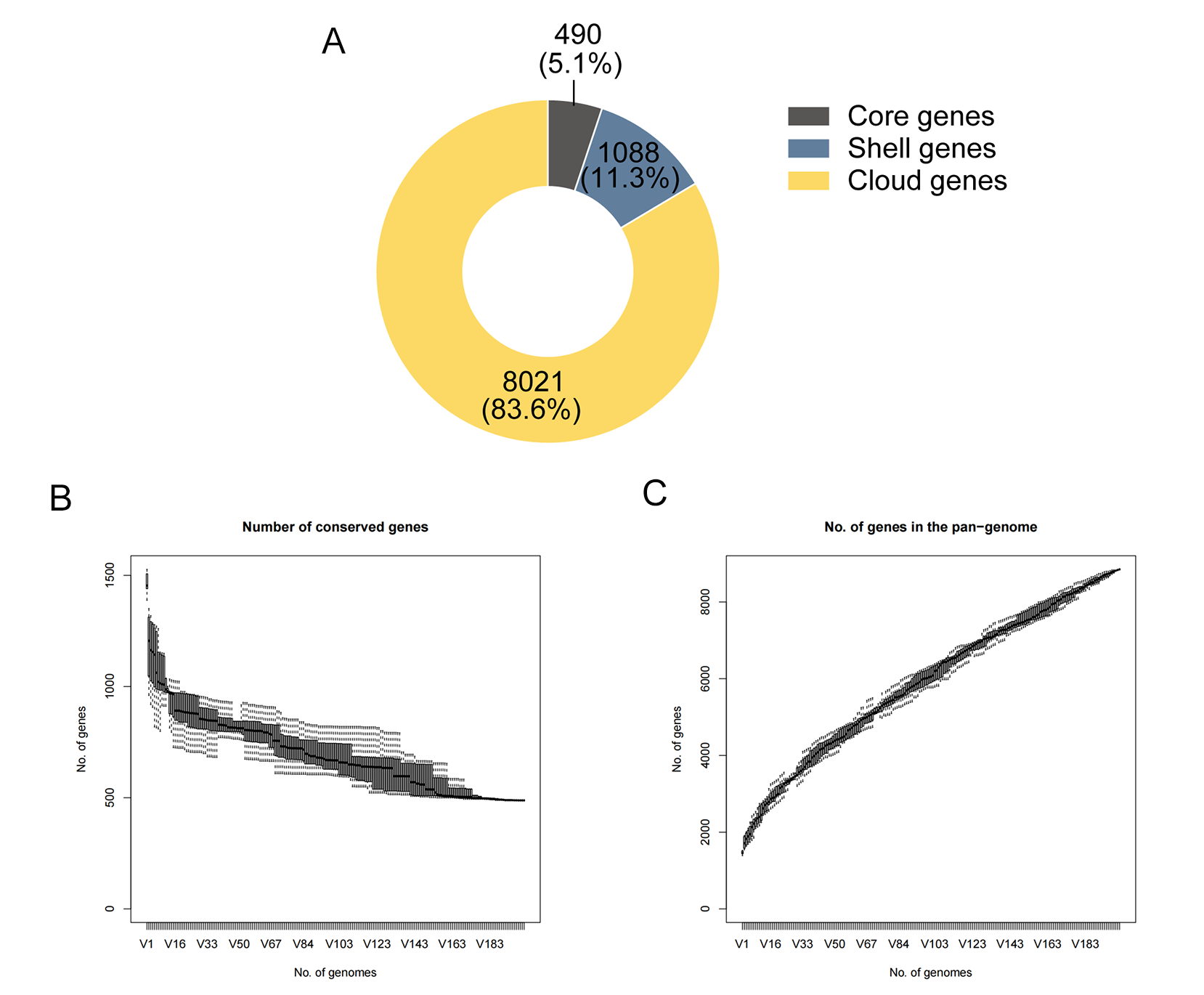

Supplement: Supplementary Figure S1.tif [file KVIR_A_2481503_SM2207.tif]
