# Supplementary material for: Assessment for antibiotic resistance in Helicobacter pylori: A practical and interpretable machine learning model based on genome-wide genetic variation
Source: Virulence. 2025 Mar 21;16(1):2481503. doi: 10.1080/21505594.2025.2481503 (PMC11934168; doi:10.1080/21505594.2025.2481503)
Supplement: Supplementary Table S2.docx [file KVIR_A_2481503_SM2206.docx]

**Supplemebtary Table S2. Association of antibiotic resistance and clinical phenotypes in 276 *H.pylori* strains**

| **Clinical phenotypes** | Total, n(%) | MTZ | | CLR | | LEV | | AMX | | MDR | |
| --- | --- | --- | --- | --- | --- | --- | --- | --- | --- | --- | --- |
| **Age** | 276(%) | Resistant | Susceptible | Resistant | Susceptible | Resistant | Susceptible | Resistant | Susceptible | Resistant | Non-MDR |
| **19-50** | 140(50.7%) | 102(37.0%) | 38(13.7%) | 46(16.7%) | 94(34%) | 52(18.8%) | 88(31.9%) | 17(6.2%) | 123(44.6%) | 29(10.5%) | 111(40.2%) |
| **51-87** | 136(49.3% | 91(33.0%) | 45(16.3%) | 38(13.8%) | 98(35.5%) | 48(17.4%) | 88(31.9%) | 8(2.9%) | 128(46.4%) | 19(6.9%) | 117(42.4%) |
| ***P* value** |  | 0.282 | | 0.375 | | 0.749 | | 0.07 | | 0.139 | |
| **Gender** |  |  |  |  |  |  |  |  |  |  |  |
| **Female** | 117(42.4%) | 83(30.1%) | 34(12.3%) | 40(14.5%) | 77(27.9%) | 51(18.5%) | 66(23.9%) | 8(2.9%) | 109(39.5%) | 26(9.4%) | 91(33.0%) |
| **Male** | 159(57.6%) | 110(39.9%) | 49(17.7%) | 44(15.9%) | 115(41.7%) | 49(17.8%) | 110(39.8%) | 17(6.2%) | 142(51.4%) | 22(8.0%) | 137(49.6%) |
| ***P* value** |  | 0.753 | | 0.245 | | 0.029 | | 0.270 | | 0.069 | |

Abbreviations: MTZ, metronidazole; CLR, clarithromycin; LEV, levofloxacin; AMX, amoxicillin; MDR, multidrug-resistant.
